# Porcine Deltacoronavirus Nsp13 Suppresses the Assembly of the MAVS‐TBK1‐IRF3 Complex and IRF9 Nuclear Translocation

**DOI:** 10.1155/tbed/3676642

**Published:** 2026-07-20

**Authors:** Ying Wang, Shijin Lan, Zhenghui Fang, Shixing Yang, Xiaochun Wang, Quan Shen, Yuwei Liu, Ping Wu, Chenglin Zhou, Wen Zhang, Likai Ji

**Affiliations:** ^1^ School of Medicine, Jiangsu University, Zhenjiang 212013, China, ujs.edu.cn; ^2^ Clinical Laboratory Center, The Affiliated Taizhou People’s Hospital of Nanjing Medical University, Taizhou 225300, China, njucm.edu.cn

**Keywords:** IFN-I, IRF9, PDCoV Nsp13, TBK1

## Abstract

Porcine deltacoronavirus (PDCoV) is an emerging enteric coronavirus that causes substantial morbidity in swine and may pose a zoonotic risk because of its cross‐species transmission potential. Type I interferon (IFN‐I) signaling is central to antiviral defense, initiated through the mitochondrial antiviral signaling (MAVS)–TBK1–IFN regulatory factor 3 (IRF3) axis and executed by the downstream JAK–STAT1–STAT2–IRF9 (ISGF3) pathway; however, how PDCoV circumvents both the induction and effector arms of this cascade remains incompletely understood. Here, we identify PDCoV nonstructural protein 13 (Nsp13) as a potent antagonist of IFN‐I responses. Ectopic expression of Nsp13 markedly reduces IFN‐β production and the expression of IFN‐stimulated genes (ISGs, such as ISG56 and CXCL10). Mechanistically, Nsp13 directly binds the C‐terminal domain (CTD) of TBK1 and competitively disrupts TBK1 interactions with IRF3 and MAVS. In addition, Nsp13 preferentially binds IRF9 and impairs its nuclear translocation, thereby inhibiting IFN‐α–induced signaling. Notably, PDCoV Nsp13 exhibits a host‐target binding profile similar to that of SARS‐CoV‐2 Nsp13, yet it does not alter TBK1 ubiquitination or protein stability. Collectively, these findings reveal a dual‐layer immune evasion strategy whereby PDCoV Nsp13 suppresses both IFN induction and downstream signaling, and highlighting Nsp13 as a potential target for antiviral intervention.

## 1. Introduction

Porcine deltacoronavirus (PDCoV) belongs to the family Coronaviridae and the genus *Deltacoronavirus* [1, 2]. Since its initial identification in 2012, PDCoV has spread widely among swine herds across Asia and North America [3]. The virus primarily infects intestinal epithelial cells, causing severe diarrhea, vomiting, and high mortality in neonatal piglets, resulting in substantial economic losses to the global swine industry [2–4]. Notably, in 2021, PDCoV was detected in children with diarrhea in Haiti, and PDCoV‐specific antibodies were found in human serum, indicating the potential for zoonotic transmission [5]. As a newly emerging potentially zoonotic pathogen, there is an urgent need to strengthen prevention strategies and elucidate its pathogenic and immune evasion mechanisms.

Coronaviruses have evolved multiple strategies to evade host innate immune responses, utilizing both structural and nonstructural proteins (Nsps). A central and conserved mechanism of immune antagonism among coronaviruses is disruption of the type I interferon (IFN‐I) signaling cascade. For instance, SARS‐CoV‐2 Nsp3 inhibits IFN‐I production via direct cleavage of IFN regulatory factor 3 (IRF3) [6, 7], whereas the MERS‐CoV M protein impairs IRF3 activation by suppressing TBK1‐dependent phosphorylation [8]. PDCoV Nsp5 cleaves STAT2 and the NF‐κB essential modulator, NEMO, through its protease activity, thereby antagonizing IFN‐I responses [9, 10]. PDCoV Nsp10 also suppresses IFN‐β production by interfering with IRF3 and NF‐κB activation [11, 12]. SARS‐CoV‐2 Nsp13 inhibits IFN‐I responses by interacting with IRF3, disrupting its activation, and recruiting TBK1 for p62‐dependent autophagic degradation [13]. Current studies have shown that PDCoV Nsp13 is a multifunctional helicase with ATP‐dependent 5′→3′ unwinding and ATP‐independent annealing [14, 15]. Unwinding is unaffected by Mg^2+^ (0.5–6.0 mmol/L) or pH (4–9) but is inhibited by high Nsp13 (≥80 nmol/L) [14]. ADP enhances annealing, whereas ATP suppresses it [15]. Nsp13 unwinds dsDNA better than dsRNA, requires a 5′ overhang ≥4 nt, and efficiency decreases with longer overhangs or duplex regions [14, 16]. Domain deletions show that ZBD loss reduces unwinding; further removal of the stalk and/or 1B abolishes unwinding but not ATPase activity, indicating that these domains are essential for duplex separation [16, 17]. However, the immunomodulatory role of PDCoV Nsp13 remains poorly understood.

Activation of host IFN‐I responses is initiated by recognition of viral RNA by pattern recognition receptors (PRRs), such as RIG‐I and MDA5 [18]. These PRRs engage mitochondrial antiviral signaling (MAVS) protein, which subsequently recruits and activates TANK‐binding kinase 1 (TBK1) to form a signalosome complex with adaptor proteins, including TRAF3 and NEMO [18]. TBK1 activation also depends on K63‐linked polyubiquitination, which promotes its oligomerization and kinase activity [19, 20]. Activated TBK1 phosphorylates IRF3, leading to IRF3 dimerization and nuclear translocation and ultimately driving IFN‐β transcription [21, 22]. Following IFN‐β production and secretion, IFN‐I bind the IFN‐α/β receptor (IFNAR1/2) and trigger the JAK–STAT signaling cascade, in which receptor‐associated JAK1 and TYK2 phosphorylate STAT1 and STAT2. Phosphorylated STAT1 and STAT2, together with IRF9, assemble into the ISGF3 complex, which translocates into the nucleus and induces a broad array of IFN‐stimulated genes (ISGs), thereby establishing an antiviral state. Thus, both the MAVS–TBK1–IRF3 module and the downstream JAK–STAT1–STAT2–IRF9 axis are central to antiviral signaling propagation, and disruption at either level impairs IFN‐I signaling and host immune responses [23–28].

PDCoV infection has been shown to suppress IFN‐β production, yet the underlying mechanisms of immune evasion remain unclear. In this study, we investigate PDCoV Nsp13 as a potential antagonist of IFN‐I signaling and uncover a mechanism by which it suppresses both IFN induction and downstream signaling. These findings advance our understanding of PDCoV immune evasion and provide a rationale for targeting virus–host interfaces to enhance antiviral defenses.

## 2. Materials and Methods

### 2.1. Antibodies and Reagents

We obtained the following antibodies and reagents for this study: GAPDH antibody, horseradish peroxidase (HRP)‐conjugated anti‐mouse IgG antibody, and HRP‐conjugated anti‐rabbit IgG antibody (all from Abclonal, Wuhan, China). Anti‐MYC tag antibody (Cat. No. 2276S), anti‐Flag tag antibody (Cat. No. D6W5B), anti‐HA tag antibody (Cat. No. H6908), phospho‐IRF3 (p‐IRF3) antibody (Cat. No. 29047T), total IRF3 antibody (Cat. No. 4302T), phospho‐TBK1 (p‐TBK1) antibody (Cat. No. 5483T), and total TBK1 antibody (Cat. No. 3504T) were purchased from Sigma–Aldrich (St. Louis, MO, USA). All antibodies were used according to the manufacturer’s instructions.

### 2.2. Plasmids and Transfection

Plasmids were constructed using a one‐step homologous recombination cloning kit (Cat. No. C112‐02, Vazyme Biotech, Nanjing, China) according to the manufacturer’s protocol. For plasmid transfection, cells at 80%–90% confluency were transfected using a liposome‐based transfection reagent (Cat. No. C0526, Beyotime, Shanghai, China). The plasmids used in this study, including those encoding TBK1, MAVS, IRF3, IRF9, and related constructs, were generated following the procedures described in our previous work [29–31]. For amplification of PDCoV Nsp13, the following primers were used: PF, 5′‐GCCAGCGGTGTTTGTGTGG‐3′; PR, 5′‐TTACTGAAGCTGTGAATCAATAGATTCA‐3′; shRNA‐762: CTCTGTTGGTGCGTCTTATTATTCAAGAGATAATAAGACGCACCAACAGAGTTTTTTCTCGAG; shRNA‐1023: GTTTCTATGTTGACCAATTATTTCAAGAGAATAATTGGTCAACATAGAAACTTTTTTCTCGAG.

### 2.3. Cell Culture and Virus

HEK‐293T cells (ATCC) were cultured in high‐glucose Dulbecco’s Modified Eagle Medium (DMEM; Cat. No. E600003‐0500, Sangon Biotech, Shanghai, China) supplemented with 10% fetal bovine serum (FBS; Cat. No. F0193, Sigma–Aldrich, St. Louis, MO, USA) and 100 U/mL penicillin–streptomycin. LLC‐PK1 cells, obtained from Tongling Shan (Shanghai Veterinary Research Institute, Shanghai, China), were maintained in Minimal Essential Medium (MEM; Cat. No. 11095080, Gibco, USA), supplemented with 10% FBS and 100 U/mL penicillin–streptomycin. All cells were cultured at 37°C in a humidified incubator with 5% CO_2_. For transfection, HEK‐293T cells were transfected using the Lipo6000 reagent (Biotin, China) according to the manufacturer’s instructions.

### 2.4. Co‐IP Assay

At 24 h posttransfection, the cells were lysed in radioimmunoprecipitation assay (RIPA) lysis buffer. The lysates were centrifuged, and the supernatants were incubated with pre‐equilibrated Anti‐Flag affinity gel (Cat. No. P2282, Beyotime, Shanghai, China) or Anti‐HA affinity gel (Cat. No. P2287, Beyotime) at 4°C for 4 h on a rotating mixer. After incubation, the beads were washed three times with 1× Tris‐buffered saline (TBS), and bound proteins were eluted by adding 5× SDS‐PAGE loading buffer. The samples were then boiled and subjected to western blot analysis.

### 2.5. Western Blot Assay

Cells were first washed with cold phosphate‐buffered saline (PBS) and lysed on ice using RIPA buffer (Cat. No. P0013D, Beyotime, Shanghai, China), supplemented with protease inhibitors (Cat. No. SB‐WB016, Share‐bio, China) for 15 min. After lysis, 5× SDS‐PAGE loading buffer (Cat. No. 20315ES05, YEASEN, Shanghai, China) was added to the samples, which were then boiled at 100°C for 15 min. Proteins were separated by SDS‐PAGE and transferred onto a PVDF or nitrocellulose membrane. The membrane was blocked with 5% skim milk (Cat. No. 36120ES76, YEASEN) for 120 min at room temperature and then incubated overnight at 4°C with the primary antibody. Following three washes with TBS containing 0.1% Tween‐20 (TBST), the membrane was incubated with the appropriate HRP‐conjugated secondary antibody for 60 min at room temperature. After additional TBST washes, protein signals were detected using an enhanced chemiluminescence (ECL) reagent (Cat. No. SB‐WB012, Share‐bio).

### 2.6. Immunofluorescence Assay

Cells were fixed at 24 h posttransfection with 4% paraformaldehyde (PFA). The fixed cells were then permeabilized with 0.1% Triton X‐100 in PBS. After washing three times with PBS, cells were blocked with 3% bovine serum albumin (BSA) in PBS at 37°C for 1 h. Primary antibodies (anti‐HA, anti‐Flag, and anti‐IRF3; Cell Signaling Technology (CST); 1:500) were diluted in blocking buffer (3% BSA in PBS) and incubated with the cells at 37°C for 4 h. After washing three times with PBS, the cells were incubated with fluorophore‐conjugated secondary antibodies (Beyotime; Alexa Fluor 488‐ or 555‐conjugated; 1:500) for 1 h at 37°C. Nuclei were counterstained with 4′,6‐diamidino‐2‐phenylindole (DAPI) in PBS. Samples were mounted and imaged using a confocal microscope (Leica TCS SP5). Images were acquired using identical laser power and detector settings for all experimental groups. Scale bars: 10 μm.

### 2.7. Luciferase Assay

Dual‐luciferase reporter assays were performed in HEK‐293T cells. Cells were maintained in DMEM supplemented with 10% fetal bovine serum at 37°C with 5% CO_2_ and seeded in 24‐well plates the day before transfection to reach ~70%–80% confluence. Cells were cotransfected with a firefly luciferase reporter plasmid, a Renilla luciferase plasmid as an internal control, and the indicated expression plasmids (or empty vector) using the Lipo6000 transfection reagent (Beyotime, Cat. No. C0526) according to the manufacturer’s instructions. Per well, 5 ng firefly reporter and 10 ng Renilla plasmid were used; the remaining DNA consisted of the indicated expression plasmids and empty vector. For all conditions, the total amount of plasmid DNA per well was adjusted to 50 ng by supplementing with an empty vector.

Where indicated, innate immune signaling was stimulated as follows: (i) poly (I:C) was cotransfected and cells were harvested at 24 h posttransfection; (ii) SeV stimulation was performed at 24 h posttransfection and cells were harvested 12 h after SeV treatment; (iii) IFN‐α stimulation was performed at 24 h posttransfection and cells were harvested 6 h after IFN‐α treatment; and (iv) for pathway activation by coexpression of RIG‐I, MDA5, MAVS, or TBK1, the indicated activators were cotransfected and cells were harvested at 24 h posttransfection. Cells were lysed, and luciferase activities were measured using a dual‐luciferase reporter assay kit (Vazyme, Cat. No. DL101‐01) according to the manufacturer’s instructions. Firefly and Renilla luciferase activities were recorded sequentially on a luminometer. Relative luciferase activity (arbitrary units) was calculated by normalizing firefly luciferase activity to Renilla luciferase activity (Firefly/Renilla). Data represent the mean ± SD from triplicate wells, and each experiment was repeated at least three times independently.

### 2.8. RT‐qPCR

Total RNA was extracted from cells using the TRIzol reagent (Thermo Fisher Scientific, USA; Cat. No. 15596026CN) according to the manufacturer’s instructions. RNA was reverse‐transcribed into complementary DNA (cDNA) using HiScript III RT SuperMix for qPCR (+gDNA wiper) (Vazyme Biotech, Nanjing, China; Cat. No. R323‐01) following the manufacturer’s protocol. Quantitative real‐time PCR (RT–qPCR) was performed using the MagicSYBR Mixture (CWBIO, Beijing, China; Cat. No. CW3008M) on a Bio‐Rad CFX96 real‐time PCR system. The cycling conditions were 95°C for 30 s, followed by 40 cycles of 95°C for 10 s and 60°C for 30 s, followed by melt‐curve analysis to confirm amplicon specificity. Relative mRNA levels were calculated using the 2^−ΔΔCt^ method and normalized to *GAPDH*. Data represent the mean ± SD from triplicate technical reactions and at least three independent biological replicates. Primers for *ISG56*, *CXCL10*, *IRF3*, and *IFIT1* have been described previously [10, 32].

### 2.9. Statistical Analysis

Data from three independent experiments were expressed as means ± standard deviations. Significance was determined with a two‐tailed Student’s *t*‐test to analyze the differences in multiple groups (≥3). *p*‐Values of <0.05 were considered statistically significant.

## 3. Results

### 3.1. PDCoV Nsp13 Inhibits IFN‐I Pathway Signaling and Promotes Viral Replication

**Figure 1:**
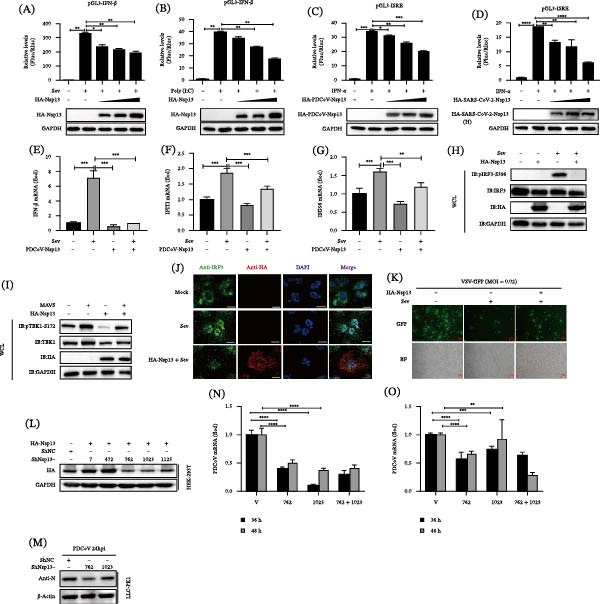
Nsp13 inhibits IFN‐I signaling and promotes viral replication. (A, B) HEK‐293T cells were cotransfected with pIFN‐β‐Luc, pRL‐TK, and increasing amounts of Nsp13 expression plasmid (200, 400, and 600 ng). At 24 h posttransfection, cells were stimulated with 500 U/mL SeV (A) or 100 ng/mL poly (I:C) (B). Luciferase activity was measured 24 h later. Firefly luciferase activity was normalized to Renilla luciferase activity. (C, D) Dual‐luciferase reporter assay using PDCoV Nsp13 and SARS‐CoV‐2 Nsp13 at different concentrations (300, 600, and 900 ng). (E–G) Relative mRNA expression of ISG56, CXCL10, and IFIT1 in SeV‐stimulated HEK‐293T cells with or without Nsp13 overexpression was analyzed by qRT‐PCR. GAPDH served as an internal control. (H, I) Western blot analysis of TBK1 and IRF3 phosphorylation. Whole‐cell lysates were collected at 24 h and probed for p‐TBK1 (S172), TBK1, p‐IRF3 (S396), and total IRF3. (J) HEK‐293T cells were transfected with PDCoV Nsp13 (or empty vector) together with IRF3 and then stimulated with SeV. Indirect immunofluorescence and confocal microscopy showed predominantly nuclear IRF3 in control cells, whereas IRF3 remained largely cytoplasmic in Nsp13‐expressing cells. Nuclei were counterstained with DAPI. (K) HEK‐293T cells transfected with HA‐Nsp13 were infected with VSV‐GFP after SeV stimulation. GFP fluorescence was captured by fluorescence microscopy and quantified using ImageJ. Scale bar = 10 μm. All data represent mean ± SD of three independent experiments.  ^∗^ *p* < 0.05;  ^∗∗^ *p* < 0.01;  ^∗∗∗^ *p* < 0.001; ns, not significant (one‐way ANOVA). (L) Screening of five shRNAs targeting PDCoV Nsp13 in LLC‐PK1 cells. (M–O) Effect of Nsp13 knockdown on PDCoV replication. LLC‐PK1 cells stably expressing sh762, sh1023, or sh‐Ctrl were infected with PDCoV (MOI = 0.1, 24 h). Data are shown as mean ± SD (*n* = 3).  ^∗∗^ *p* < 0.01, ^∗∗∗^ *p* < 0.001 versus sh‐Ctrl.

To investigate the role of PDCoV Nsp13 in regulating the IFN‐I pathway, we employed a dual‐luciferase reporter assay in HEK‐293T cells. Nsp13 expression significantly suppressed Sendai virus (SeV)‐ and poly (I:C)‐induced activation of the IFN‐β promoter in a dose‐dependent manner (Figure 1A,B). Consistently, similar results were obtained in PK1 cells (Figure S1A,B). Functionally, both PDCoV Nsp13 and SARS‐CoV‐2 Nsp13 dose‐dependently inhibited ISRE‐driven reporter activity, whereas the empty vector had minimal effects (Figure 1C,D). Together, these results identify Nsp13 as a potent inhibitor of RLR–IRF3–IFN signaling and support Nsp13‐mediated suppression of downstream IFN‐I responses as a conserved immune‐evasion strategy among coronaviruses.

To evaluate the broader inhibitory impact of Nsp13 on ISGs, RT‐qPCR revealed marked downregulation of *IFIT1*, *CXCL10*, and *ISG56* transcripts (Figure 1E–G). The same RT‐qPCR analysis was also performed in PK1 cells, yielding consistent downregulation (Figure S1C–E). Given the essential roles of IRF3 and TBK1 phosphorylation in triggering IFN‐I production [25, 33], we assessed the phosphorylation status and found that Nsp13 significantly reduced phosphorylation at Ser396 of IRF3 and Ser172 of TBK1 (Figure 1H,I), suggesting that Nsp13 interferes with early stages of IFN signaling activation. Furthermore, Nsp13 inhibited JAK1 Tyr1022/1023 and STAT1 Tyr701 phosphorylation (Figure S1F). In SeV‐pretreated HEK‐293T cells, Nsp13 overexpression reversed the SeV‐induced antiviral state and facilitated replication of GFP‐tagged vesicular stomatitis virus (VSV‐GFP), as evidenced by enhanced GFP expression (Figure 1K). Moreover, two shRNA constructs (sh762 and sh1023) targeting PDCoV Nsp13 exhibited the highest knockdown efficiency (Figure 1L). Both the protein and mRNA levels of PDCoV N protein were significantly reduced in the sh762‐ and sh1023‐expressing LLC‐PK1 cells with PDCoV infection (Figure 1M–O). These results demonstrate that knockdown of Nsp13 effectively suppresses PDCoV replication, indicating that endogenous Nsp13 plays a critical role in the life cycle of PDCoV. Collectively, these findings indicate that PDCoV Nsp13 compromises intrinsic antiviral defenses by inhibiting the IFN‐I pathway activation, thereby creating a permissive environment for viral replication.

### 3.2. PDCoV Nsp13 Suppresses RLR–IRF3–IFN Signaling

**Figure 2:**
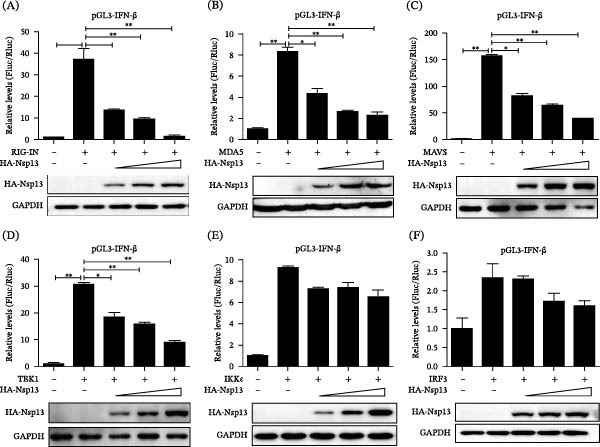
Nsp13 suppresses IFN‐β promoter activation induced by RLR signaling components in a dose‐dependent manner. (A–F) HEK‐293T cells were cotransfected with pIFN‐β‐Luc, pRL‐TK, and plasmids encoding RIG‐IN (A), MDA5 (B), MAVS (C), TBK1 (D), IKKε (E), or IRF3 (F), along with increasing amounts (0, 100, 200, 300 ng) of Nsp13 expression plasmid. Luciferase assays were performed 24 h posttransfection. Data are shown as mean ± SD from three independent experiments.  ^∗^ *p* < 0.05;  ^∗∗^ *p* < 0.01;  ^∗∗∗^ *p* < 0.001 (one‐way ANOVA).

To pinpoint the step(s) of IFN‐β induction targeted by Nsp13, dual‐luciferase reporter assays were used to systematically assess key components of the RIG‐I‐like receptor (RLR) pathway, including RIG‐IN, MDA5, MAVS, TBK1, IKKε, and IRF3 [18, 26]. Increasing amounts of Nsp13 (100, 200, and 300 ng) dose‐dependently suppressed IFN‐β promoter activation driven by RIG‐IN, MDA5, MAVS, TBK1, and IRF3 (Figure 2A–F). Consistent with the marked reduction in IRF3 nuclear translocation upon Nsp13 expression (Figure 1J), these data suggest that Nsp13 interferes with IFN‐β induction by targeting IRF3 or an upstream signaling component.

### 3.3. PDCoV Nsp13 Interacts With TBK1

**Figure 3:**
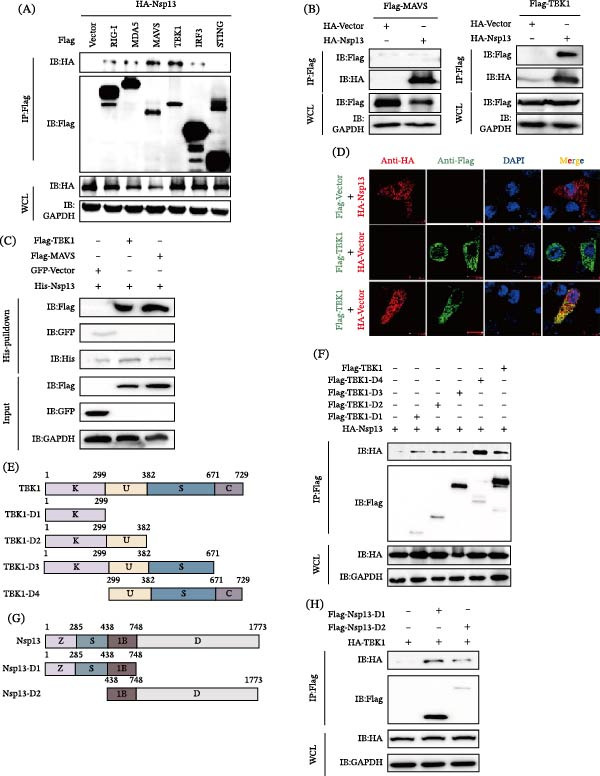
TBK1‐Nsp13 interaction is mediated by the CTD and the 1B domain. (A, B) Co‐immunoprecipitation (Co‐IP) of HA‐Nsp13 with Flag‐tagged signaling proteins (RIG‐I, MDA5, MAVS, TBK1, IRF3, STING) expressed in HEK‐293T cells. Flag‐tagged proteins were immunoprecipitated with anti‐Flag affinity gel and probed with anti‐HA. (C) His‐pulldown assay using purified His‐Nsp13 protein incubated with lysates from Flag‐TBK1 or GFP‐vector‐expressing HEK‐293T cells. (D) Confocal microscopy of LLC‐PK1 cells transfected with HA‐Nsp13 and Flag‐TBK1 for 24 h. Cells were immunostained with anti‐HA (red), anti‐Flag (green), and DAPI (blue). Colocalization was observed in the cytoplasm. Scale bar = 10 μm. (E, F) HEK‐293T cells were cotransfected with HA‐Nsp13 and full‐length or truncated TBK1 constructs (D1–D4). Co‐IP was performed using anti‐Flag. The strongest binding was observed with TBK1‐D4 containing the CTD. (G, H) HEK‐293T cells were transfected with Flag‐TBK1 and full‐length or truncated Nsp13 constructs (Nsp13‐D1, Nsp13‐D2). At 24 h posttransfection, Co‐IP was performed using anti‐Flag antibody, and coprecipitated Nsp13 was detected by western blotting. Both truncates containing the 1B domain retained TBK1‐binding capacity, suggesting that the 1B domain is critical for interaction.

To test this hypothesis, we conducted co‐immunoprecipitation (Co‐IP) assays by coexpressing Nsp13 with individual RLR signaling proteins in HEK‐293T cells. In addition to the canonical RLR pathway components (RIG‐IN, MDA5, MAVS, TBK1, and IRF3), we also tested STING, a central adaptor in the DNA‐sensing cGAS‐STING pathway, as a specificity control. Among these, only TBK1 was consistently coprecipitated with Nsp13 using different affinity resins (Figure 3A,B), suggesting a specific interaction. To further validate this finding, we performed a His‐pulldown assay using bacterially expressed His‐tagged Nsp13. The result confirmed that Nsp13 could coprecipitate overexpressed TBK1 from HEK‐293T cell lysates (Figure 3C). Additionally, immunofluorescence microscopy in LLC‐PK1 cells revealed colocalization of Nsp13 and TBK1 in the cytoplasm, further supporting their physical association (Figure 3D).

To map the interaction interfaces between Nsp13 and TBK1, domain‐truncation analyses were performed for both proteins. Four TBK1 truncation mutants (TBK1‐D1–D4) were generated as previously described [34]: TBK1‐D1 contains the kinase domain (KD), TBK1‐D2 comprises KD plus the ubiquitin‐like domain (ULD), TBK1‐D3 includes KD, ULD, and the scaffold dimerization domain (SDD), and TBK1‐D4 consists of ULD, SDD, and the C‐terminal domain (CTD) (Figure 3E). HA‐tagged Nsp13 was coexpressed with Flag‐tagged wild‐type TBK1 or each TBK1 truncation mutant in HEK‐293T cells, followed by anti‐Flag Co‐IP. Nsp13 was associated with all TBK1 truncation mutants, with the strongest binding observed for TBK1‐D4 (Figure 3F), suggesting a preferential association with the CTD‐containing region. Notably, the CTD has been implicated in conformational regulation of TBK1 activation and maintenance of signaling complex integrity [25, 34], supporting the TBK1 CTD as a key region engaged by Nsp13.

In parallel, Nsp13 domain mapping was conducted (Figure 3G). PDCoV Nsp13 is a virus‐encoded helicase composed of the zinc‐binding domain (ZBD), stalk domain, 1B domain, and helicase core [14, 17]. Two Nsp13 truncation mutants (Nsp13‐D1 and Nsp13‐D2) were constructed and coexpressed with Flag‐tagged TBK1 in HEK‐293T cells. Anti‐Flag Co‐IP followed by immunoblotting revealed that both Nsp13‐D1 and Nsp13‐D2 were coprecipitated with TBK1 (Figure 3H), implicating that the shared 1B domain is required for TBK1 binding. Collectively, these results suggest that Nsp13 engages TBK1 primarily through the Nsp13 1B domain and preferentially associates with the TBK1 CTD‐containing region, providing a molecular basis for the Nsp13‐mediated modulation of TBK1‐centered antiviral signaling.

### 3.4. PDCoV Nsp13 Disrupts the MAVS‐TBK1‐IRF3 Complex Conformation

TBK1 is a serine/threonine kinase that phosphorylates and activates IRF3 [26]. Building upon the previously identified direct interaction between Nsp13 and TBK1, we further explored whether Nsp13 interferes with TBK1’s binding to its downstream signaling partners—IRF3 and MAVS—through competitive (steric) mechanisms.

**Figure 4:**
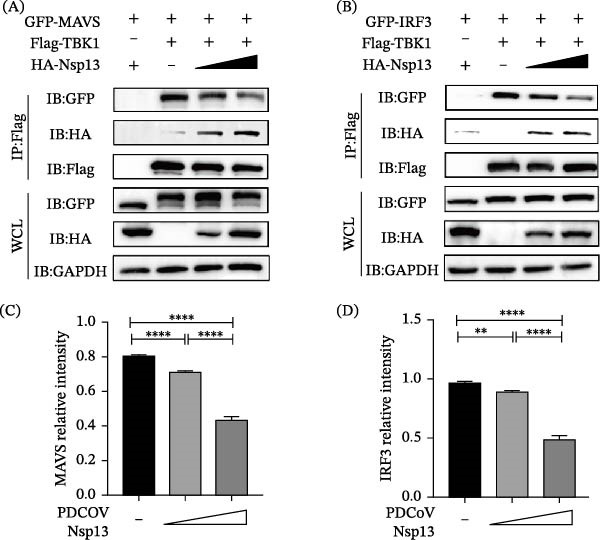
Nsp13 competitively disrupts TBK1‐IRF3 and TBK1‐MAVS complex formation. (A, B) HEK‐293T cells were cotransfected with Flag‐TBK1, GFP‐IRF3 (A) or GFP‐MAVS (B), and increasing amounts of HA‐Nsp13 (1.0, 2.0 µg). Co‐IP was performed using anti‐Flag affinity gel. Immunoblots revealed that increasing levels of Nsp13 impaired the TBK1‐IRF3 and TBK1‐MAVS interactions in a dose‐dependent manner. Data are representative of three independent experiments. (C, D) Densitometric quantification of the western blot results shown in (A–B). Data are presented as mean ± SD from three independent experiments.  ^∗∗^ *p* < 0.01, and  ^∗∗∗∗^ *p* < 0.0001.

To test this, HEK‐293T cells were cotransfected with plasmids encoding Flag‐tagged TBK1, GFP‐tagged IRF3 (or GFP‐tagged MAVS), and increasing amounts of HA‐tagged Nsp13 (1.0, and 2.0 µg). Competitive Co‐IP assays were performed to assess how Nsp13 expression affects TBK1 association with IRF3 or MAVS. The results revealed that TBK1’s interaction with IRF3 decreased in a dose‐dependent manner as Nsp13 levels increased. Similarly, the association between TBK1 and MAVS was also significantly attenuated (Figure 4A,B).

These findings demonstrate that PDCoV Nsp13 disrupts the assembly of the antiviral signaling complex by competitively occupying the CTD of TBK1, thereby impairing its interactions with both IRF3 and MAVS. This mechanism provides a molecular basis for understanding how Nsp13 facilitates immune evasion during viral infection.

### 3.5. Differential Effects of PDCoV and SARS‐CoV‐2 Nsp13 on TBK1 Ubiquitination

To determine whether this IFN antagonism is conserved across coronaviruses, Nsp13 sequences from PDCoV and representative coronaviruses (including SARS‐CoV‐2, PEDV, HCoV‐229E, and MERS‐CoV) were compared. Multiple sequence alignments revealed substantial conservation, particularly within the helicase core and motifs required for NTP binding and hydrolysis (Figure S2A). A PDCoV Nsp13 structural model generated by SWISS‐MODEL using SARS‐CoV Nsp13 as a template superimposed well with the SARS‐CoV‐2 Nsp13 structure, indicating a conserved overall fold and domain organization (Figure S2B).

**Figure 5:**
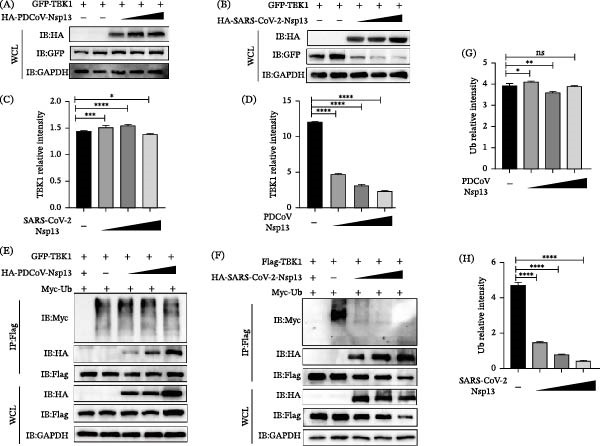
Differential effects of PDCoV and SARS‐CoV‐2 Nsp13 on TBK1 stability and ubiquitination. (A, B) HEK‐293T cells were cotransfected with GFP‐TBK1 and increasing amounts of HA‐PDCoV‐Nsp13 or HA‐SARS‐CoV‐2‐Nsp13. Whole‐cell lysates (WCL) were subjected to immunoblotting (IB) with the indicated antibodies. (C, D) Densitometric quantification of the western blot results shown in (A, B). Data are presented as mean ± SD from three independent experiments. (E, F) HEK‐293T cells were cotransfected with Flag‐TBK1, Myc‐tagged ubiquitin (Myc‐Ub), and increasing amounts of HA‐PDCoV‐Nsp13 or HA‐SARS‐CoV‐2‐Nsp13. Flag‐TBK1 was affinity‐precipitated using anti‐Flag agarose beads (co‐IP) and analyzed by immunoblotting (IB) with the indicated antibodies. GAPDH served as a loading control. (G, H) Densitometric quantification of the western blot results shown in (E, F). Data are presented as mean ± SD from three independent experiments.  ^∗^ *p* < 0.05,  ^∗∗^ *p* < 0.01, and  ^∗∗∗^ *p* < 0.001.

To compare the effects of coronavirus Nsp13 proteins on TBK1 stability and ubiquitination, we performed coexpression and Co‐IP assays in HEK‐293T cells. In the GFP‐TBK1 system, PDCoV Nsp13 had little effect on TBK1 abundance, whereas SARS‐CoV‐2 Nsp13 reduced GFP‐TBK1 levels in a dose‐dependent manner, consistent with enhanced TBK1 degradation (Figure 5A–D). We next assessed TBK1 ubiquitination using Flag‐TBK1 Co‐IP followed by detection of Myc‐tagged ubiquitin (Myc‐Ub). PDCoV Nsp13 did not markedly affect TBK1 ubiquitination as Myc‐Ub signals remained largely unchanged across increasing Nsp13 doses (Figure 5E,G). In contrast, SARS‐CoV‐2 Nsp13 caused a robust, dose‐dependent reduction in TBK1‐associated Myc‐Ub signals (Figure 5F,H), indicating potent suppression of TBK1 polyubiquitination. Together, these results indicate that although PDCoV Nsp13 retains a similar structure to other coronavirus Nsp13 proteins and can bind to TBK1, it differs in its mechanism of antagonizing TBK1‐mediated signaling.

### 3.6. PDCoV Nsp13 Interacts With JAK, STAT1, and IRF9

To determine whether coronavirus Nsp13 proteins directly target key components of the JAK1–STAT1–IRF9 module,Co‐IP assays were performed to examine interactions between Nsp13 and JAK1, STAT1, or IRF9. Expression plasmids encoding SARS‐CoV‐2 Nsp13 or PDCoV Nsp13 were cotransfected with JAK1, STAT1, or IRF9 into HEK‐293T cells. Co‐IP analyses showed that immunoprecipitation of JAK1, STAT1, or IRF9 resulted in coprecipitation of both SARS‐CoV‐2 and PDCoV Nsp13 (Figure 6A–D), whereas empty‐vector controls or immunoprecipitations using irrelevant antibodies did not yield comparable signals, supporting the specificity of these associations.

Figure 6PDCoV Nsp13 interacts with JAK1/STAT1/IRF9 and blocks IRF9 nuclear translocation. (A–D) Co‐immunoprecipitation (Co‐IP) of HA‐tagged PDCoV Nsp13 or HA‐tagged SARS‐CoV‐2 Nsp13 with JAK1 and IRF9 (Flag‐tagged) or STAT1 (GFP‐tagged) in HEK‐293T cells. Flag‐tagged proteins were affinity‐precipitated using anti‐Flag gel, and GFP‐tagged proteins were affinity‐precipitated using anti‐GFP gel, followed by immunoblotting with anti‐HA. (E–G) Confocal microscopy of Huh‐7 cells transfected with HA‐Nsp13 and Flag‐JAK1, Flag‐STAT1, and Flag‐IRF9 for 24 h. Cells were immunostained with anti‐HA (red), anti‐Flag (green), and DAPI (blue). Colocalization was observed in the cytoplasm. Scale bar = 10 μm. (H, I) Schematic representations of Flag‐tagged JAK1 (D1–D4) and STAT1 (D1–D6) truncation constructs used for co‐IP experiments. (J) HEK‐293T cells were cotransfected with HA‐Nsp13 and truncated JAK1 constructs (D1–D4). Co‐IP was performed using anti‐Flag affinity gel. (K) HEK‐293T cells were cotransfected with HA‐Nsp13 and Flag‐tagged truncated STAT1 constructs (D1–D6). Co‐IP was performed using anti‐Flag affinity gel. (L) HEK‐293T cells were transfected with PDCoV Nsp13 (or empty vector) together with IRF9 and then stimulated with IFN‐α. Indirect immunofluorescence and confocal microscopy showed predominantly nuclear IRF9 in control cells, whereas IRF9 remained largely cytoplasmic in Nsp13‐expressing cells. Nuclei were counterstained with DAPI. Scale bar = 10 μm. (M) Schematic model of PDCoV Nsp13‐mediated inhibition of type I interferon signaling.

**Figure:**
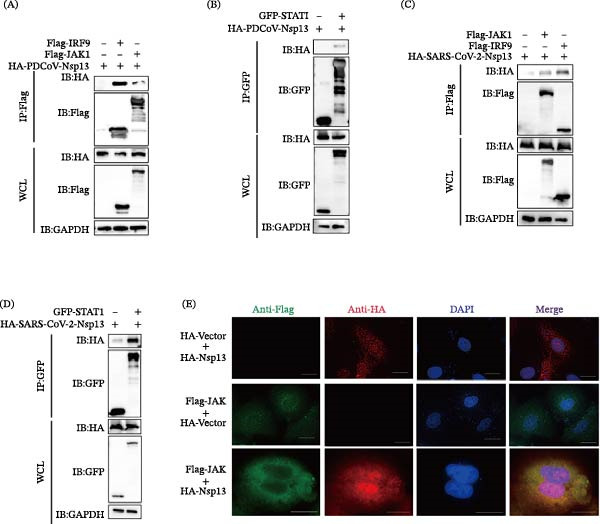

**Figure:**
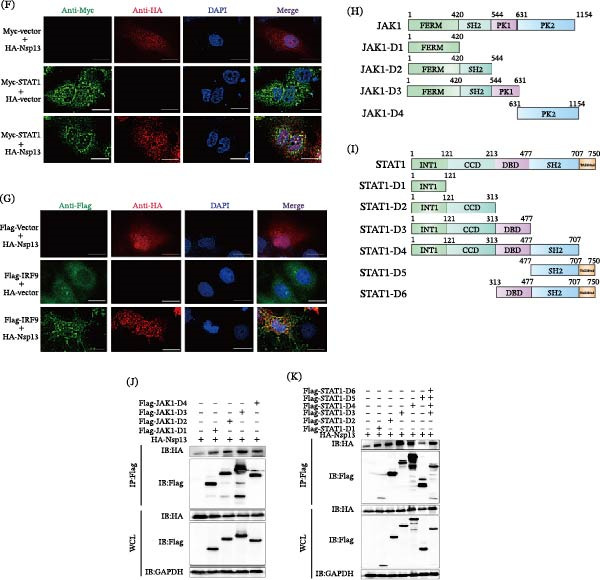

**Figure:**
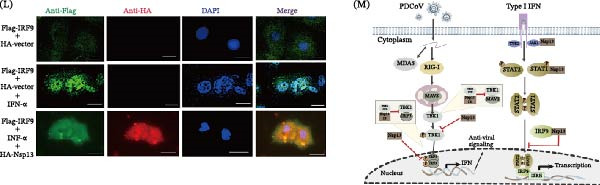

To further validate these interactions and assess their subcellular distribution, indirect immunofluorescence staining followed by confocal microscopy was conducted in cells coexpressing Nsp13 with JAK1, STAT1, or IRF9. PDCoV Nsp13 exhibited partial colocalization with JAK1, STAT1, and IRF9 in perinuclear or nuclear‐adjacent regions, with markedly increased colocalization compared with single‐expression controls (Figure 6E–G). To map the domains responsible for Nsp13 binding, we generated a series of truncated constructs of JAK1 and STAT1. JAK1 truncations (D1–D4) comprised the FERM, SH2, PK1, and PK2 domains (Figure 6H). STAT1 truncations (D1–D6) comprised the INT, CCD, DBD, SH2, and TAZ2‐binding domains (Figure 6I). Co‐IP assays using these truncations revealed that the D3 region of JAK1 (corresponding to PK1) and the D3 region of STAT1 (corresponding to DBD) exhibited the strongest binding to PDCoV Nsp13 (Figure 6J,K). These results indicate that the PK1 domain of JAK1 and the DBD domain of STAT1 serve as the primary interaction interfaces with Nsp13. Notably, Nsp13 expression reduced IFN‐α‐induced nuclear translocation of IRF9 (Figure 6L), consistent with the disruption of ISGF3‐dependent signaling.

Collectively, these data indicate that Nsp13 engages multiple components of the JAK1–STAT1–IRF9 module (JAK1, STAT1, and IRF9) and interferes with IRF9 nuclear trafficking, providing mechanistic support for Nsp13‐mediated suppression of downstream IFN‐I responses.

## 4. Discussion

IFN‐I serves as the first line of defense against virus infection, exerting both direct antiviral effects and immunomodulatory functions by inducing ISGs [18, 35, 36]. Clinical evidence indicates a strong correlation between the strength of the initial IFN‐I response and the severity of coronavirus infection [37]. In this study, we demonstrate that Nsp13 effectively suppresses IFN‐I responses by directly competing with key downstream signaling molecules for binding to the central kinase TBK1, thereby disrupting essential protein–protein interactions within the antiviral signaling cascade. These findings not only reveal a novel immunomodulatory function of PDCoV Nsp13 but also underscore the pivotal role of coronavirus Nsps in antagonizing host innate immune defenses.

Mechanistically, Nsp13 interacts directly with the CTD of TBK1 via its 1B domain. This interaction disrupts the formation of the essential TBK1–IRF3 and MAVS–TBK1 complexes, which are required for IRF3 activation and subsequent IFN‐β transcription. The disruption is dose‐dependent and consistent with steric competition, suggesting that Nsp13 binding blocks interaction interfaces on TBK1. Similar immune antagonistic mechanisms have been observed in other coronaviruses. SARS‐CoV‐2 Nsp13 directly binds to the host signaling molecule TBK1, reduces the phosphorylation of both TBK1 and IRF3, and blocks the activation of NF‐κB and IRF3‐mediated transcription of IFN‐I [38]. Additionally, the structural N protein of SARS‐CoV‐2 has been shown to interact directly with host STAT1 and STAT2 proteins, inhibiting their phosphorylation at Tyr701 and Tyr690, respectively, and preventing their nuclear translocation [39]. This competitive interference effectively suppresses downstream JAK–STAT signaling and significantly reduces IFN‐I‐induced gene expression [39]. Furthermore, the SARS‐CoV‐2 spike protein competitively binds STAT2 and interferes with the assembly of the STAT1/STAT2/IRF9 (ISGF3) complex by sequestering it in the endoplasmic reticulum, thereby blocking ISG expression [40]. Our findings expand this paradigm by demonstrating that PDCoV utilizes Nsp13—a helicase—as a direct suppressor of innate immune signaling.

A particularly intriguing observation is the differential impact of Nsp13 on TBK1 ubiquitination. SARS‐CoV‐2 Nsp13 promotes the degradation of TBK1 through p62‐dependent selective autophagy, thereby suppressing IFN production [13]. Consistent with this, our data further support this mechanism: increasing amounts of SARS‐CoV‐2 Nsp13 resulted in a gradual decrease in TBK1 protein levels, highlighting its capacity to target TBK1 for degradation (Figure 5). In contrast, PDCoV Nsp13 had no appreciable effect on TBK1 protein stability. We speculate that PDCoV Nsp13 binding to TBK1 may prioritize spatial inhibition of the MAVS‐TBK1‐IRF3 axis without altering its polyubiquitination. However, it remains unclear whether PDCoV Nsp13 modulates mono‐ubiquitination or atypical ubiquitination events not detected in our study. The possibility that Nsp13 sterically hinders the recruitment of E3 ligases or ubiquitin‐binding adaptors (e.g., OPTN and NDP52) also merits further investigation. This mechanistic difference highlights the evolutionary plasticity of coronavirus immune antagonism, suggesting that structurally homologous proteins may employ distinct molecular strategies.

However, our findings must be interpreted within the study’s limitations. Most experiments were conducted in HEK‐293T or LLC‐PK1 cells, and while these models provide valuable mechanistic insights, it is necessary to establish physiological relevance in vivo. Moreover, whether Nsp13 affects other components of the antiviral signaling network (e.g., TRAFs, STING, and NF‐κB) remains to be determined. It is also unclear whether the 1B–CTD interaction could serve as a viable therapeutic target, as blocking this interaction might also disrupt essential cellular TBK1 functions unrelated to viral immunity. Furthermore, nucleocytoplasmic fractionation assays were not performed, as the existing morphological and functional evidence is already robust and such biochemical quantification would not alter our major conclusions. Nevertheless, we acknowledge that this additional experiment would provide quantitative corroboration, and we plan to address this limitation in future studies. Most of the mechanistic experiments in this study were performed using exogenous overexpression systems. Although we obtained highly consistent and reproducible results across HEK‐293T and LLC‐PK1 cells using multiple orthogonal methods (Co‐IP, immunofluorescence, reporter assays, and qRT‐PCR), key findings—such as the TBK1‐Nsp13 interaction and Nsp13‐mediated inhibition of IRF9 nuclear translocation—await further validation in the context of authentic PDCoV infection. Moreover, whether the effects of Nsp13 on TBK1 ubiquitination and the assembly of the MAVS‐TBK1‐IRF3 signalosome are fully recapitulated during viral infection remains to be determined. Therefore, the molecular mechanisms uncovered in this study warrant consolidation and extension in future studies using PDCoV infection models.

In summary, our study uncovers a dual mechanism by which PDCoV Nsp13 suppresses IFN‐I responses: competitive binding to TBK1 that disrupts signalosome assembly and potential modulation of TBK1 ubiquitination dynamics. These findings shed light on the multifunctional nature of coronaviral helicases and lay the groundwork for future antiviral strategies targeting host–pathogen interaction interfaces.

## 5. Conclusion

In this study, we provide a comprehensive characterization of the molecular mechanisms by which PDCoV Nsp13 antagonizes the IFN‐I response. We demonstrate that PDCoV Nsp13 inhibits IFN‐β production and the expression of key ISGs, along with reduced phosphorylation of TBK1 and impaired nuclear translocation of IRF3, ultimately promoting viral replication. Mechanistically, Nsp13 interacts with the CTD of TBK1 through its 1B domain, competitively disrupting the formation of TBK1–IRF3 and TBK1–MAVS complexes. Additionally, we show that PDCoV Nsp13 disrupts the JAK–STAT1–STAT2–IRF9 signaling axis by binding directly to JAK1, STAT1, and IRF9, thereby impairing the formation of the ISGF3 complex, blocking IRF9 nuclear translocation, and inhibiting downstream ISG expression. Unlike SARS‐CoV‐2 Nsp13, PDCoV Nsp13 exhibits a limited effect on TBK1 ubiquitination, suggesting a distinct mechanism of immune interference. These findings identify PDCoV Nsp13 as a critical antagonist of both the MAVS–TBK1–IRF3 axis and the JAK–STAT1 pathway, emphasizing its role in suppressing innate immune responses and facilitating viral replication. Given its central role in immune evasion, PDCoV Nsp13 represents a potential target for antiviral intervention, and our work provides a foundation for future therapeutic strategies aimed at combating PDCoV and other coronaviruses (Figure 6M).

## Author Contributions

**Ying Wang**: methodology, data curation, writing – original draft. **Shijin Lan**: methodology, validation, writing – original draft. **Zhenghui Fang**: validation. **Shixing Yang**, **Xiaochun Wang, Quan Shen, Yuwei Liu, and Ping Wu:** supervision. **Chenglin Zhou, Wen Zhang, and Likai Ji**: supervision, writing – reviewing and editing, funding acquisition.

## Funding

This work was supported by the National Natural Science Foundation of China (Grant 32102682), the Postdoctoral Science Foundation of China (Grant 2022M721391), and the Natural Science Foundation of Higher Education of Jiangsu Province (Grant 21KJB230006).

## Ethics Statement

This article does not contain any studies with human or animal subjects performed by any of the authors.

## Conflicts of Interest

The authors declare no conflicts of interest.

## Supporting Information

Additional supporting information can be found online in the Supporting Information section.

## Supporting information


**Supporting Information** Figure S1: PDCoV Nsp13 suppresses IFN‐I signaling in PK1 cells. (A,B) Dual‐luciferase reporter assays in PK1 cells. (A) IFN‐β luciferase with SeV stimulation; (B) ISRE luciferase with IFNα stimulation. Nsp13 dose‐dependently suppressed both reporter activities. (C–E) qRT‐PCR of IFIT1 (C), CXCL10 (D), and ISG56 (E) in PK1 cells expressing Nsp13 or empty vector. Nsp13 downregulated all three ISGs, consistent with Figure 1E–G. (F) Western blot in PK1 cells. Nsp13 inhibited JAK1 (Tyr1022/1023) and STAT1 (Tyr701) phosphorylation without affecting total protein levels. Data represent three independent experiments. Figure S2: Comparative analysis of Nsp13 across coronaviruses. (A) Multiple sequence alignment of PDCoV Nsp13 with Nsp13 from other coronaviruses, including SARS‐CoV‐2, PEDV, HCoV‐229E, and MERS‐CoV. (B) Three‐dimensional structural models of PDCoV Nsp13 and SARS‐CoV Nsp13 predicted using SWISS‐MODEL.

## Data Availability

All data related to this study are included in the article. Other data can be obtained from the corresponding author if reasonably required.
