# Supplementary material for: Porcine Deltacoronavirus Nsp13 Suppresses the Assembly of the MAVS‐TBK1‐IRF3 Complex and IRF9 Nuclear Translocation
Source: Transbound Emerg Dis. 2026 Jul 20;2026:3676642. doi: 10.1155/tbed/3676642 (PMC13385975; doi:10.1155/tbed/3676642)
Supplement: Supplementary file 1 — Supporting Information Figure S1: PDCoV Nsp13 suppresses IFN‐I signaling in PK1 cells. (A,B) Dual‐luciferase reporter assays in PK1 cells. (A) IFN‐β luciferase with SeV stimulation; (B) ISRE luciferase with IFNα stimulation. Nsp13 dose‐dependently suppressed both reporter activities. (C–E) qRT‐PCR of IFIT1 (C), CXCL10 (D), and ISG56 (E) in PK1 cells expressing Nsp13 or empty vector. Nsp13 downregulated all three ISGs, consistent with Figure 1E–G. (F) Western blot in PK1 cells. Nsp13 inhibited JAK1 (Tyr1022/1023) and STAT1 (Tyr701) phosphorylation without affecting total protein levels. Data represent three independent experiments. Figure S2: Comparative analysis of Nsp13 across coronaviruses. (A) Multiple sequence alignment of PDCoV Nsp13 with Nsp13 from other coronaviruses, including SARS‐CoV‐2, PEDV, HCoV‐229E, and MERS‐CoV. (B) Three‐dimensional structural models of PDCoV Nsp13 and SARS‐CoV Nsp13 predicted using SWISS‐MODEL. [file TBED-2026-3676642-s001.docx]

**Porcine Deltacoronavirus Nsp13 Suppresses the Assembly of the MAVS-TBK1-IRF3 Complex and IRF9 Nuclear Translocation**

Ying Wang^a^, Shijin Lan^a^, Zhenghui Fang^a^, Shixing Yang^a^, Xiaochun Wang^a^, Quan Shen^a^, Yuwei Liu^a^, Ping Wu^a^, Chenglin Zhou^b^*, Wen Zhang^a^*, Likai Ji^a^*

a School of Medicine, Jiangsu University, Zhenjiang 212013, China.

b Clinical Laboratory Center, The Affiliated Taizhou People’s Hospital of Nanjing Medical University, Taizhou 225300, China.

* Address correspondence to Likai Ji, [jilikai01@ujs.edu.cn](mailto:jilikai01@ujs.edu.cn), or Wen Zhang, [zhangwen@ujs.edu.cn](mailto:zhangwen@ujs.edu.cn), or Chenglin Zhou, [18762340015@njmu.edu.cn](mailto:18762340015@njmu.edu.cn)


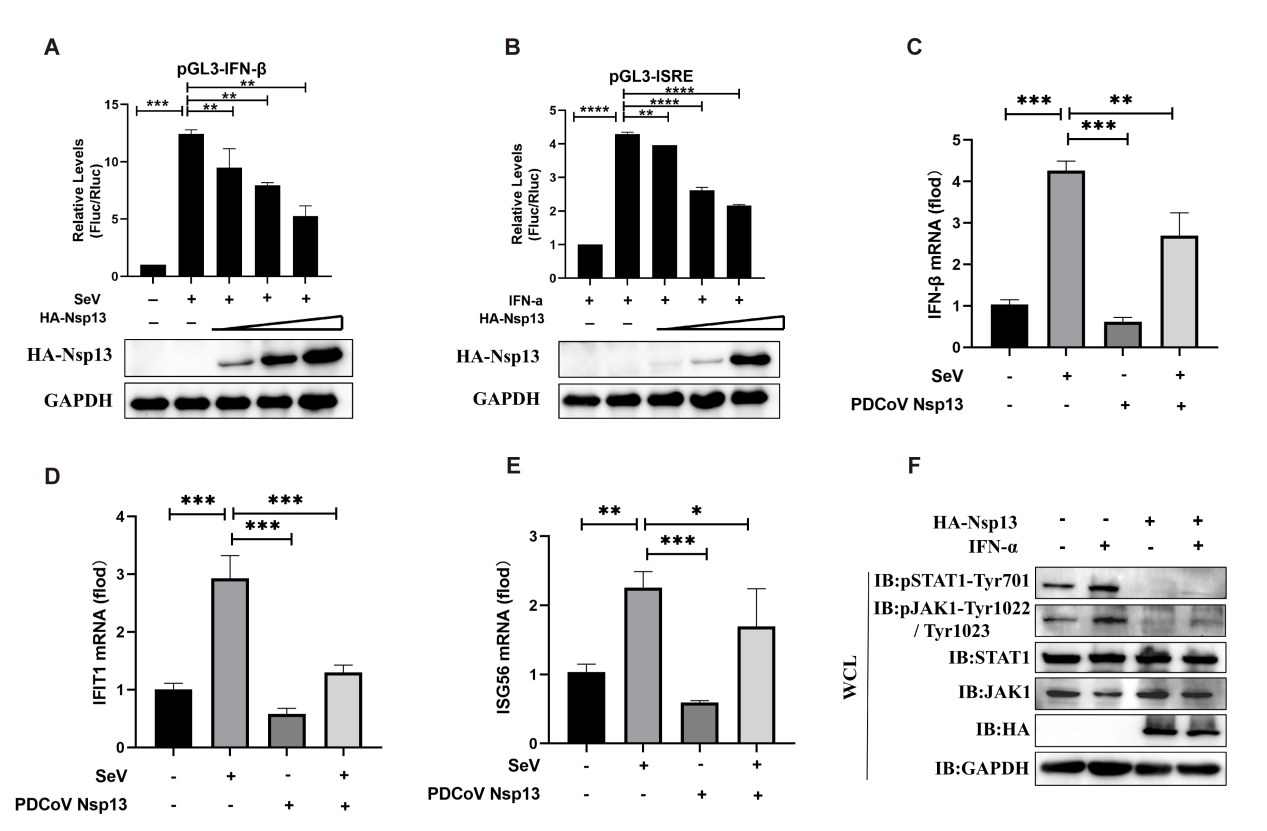


**Fig.S1: PDCoV Nsp13 suppresses IFN-I signaling in PK1 cells.**

(A–B) Dual-luciferase reporter assays in PK1 cells. (A) IFN-β luciferase with SeV stimulation; (B) ISRE luciferase with IFNα stimulation. Nsp13 dose-dependently suppressed both reporter activities.

(C–E) qRT-PCR of IFIT1 (C), CXCL10 (D), and ISG56 (E) in PK1 cells expressing Nsp13 or empty vector. Nsp13 downregulated all three ISGs, consistent with Fig. 1E–G.

(F) Western blot in PK1 cells. Nsp13 inhibited JAK1 (Tyr1022/1023) and STAT1 (Tyr701) phosphorylation without affecting total protein levels. Data represent three independent experiments.


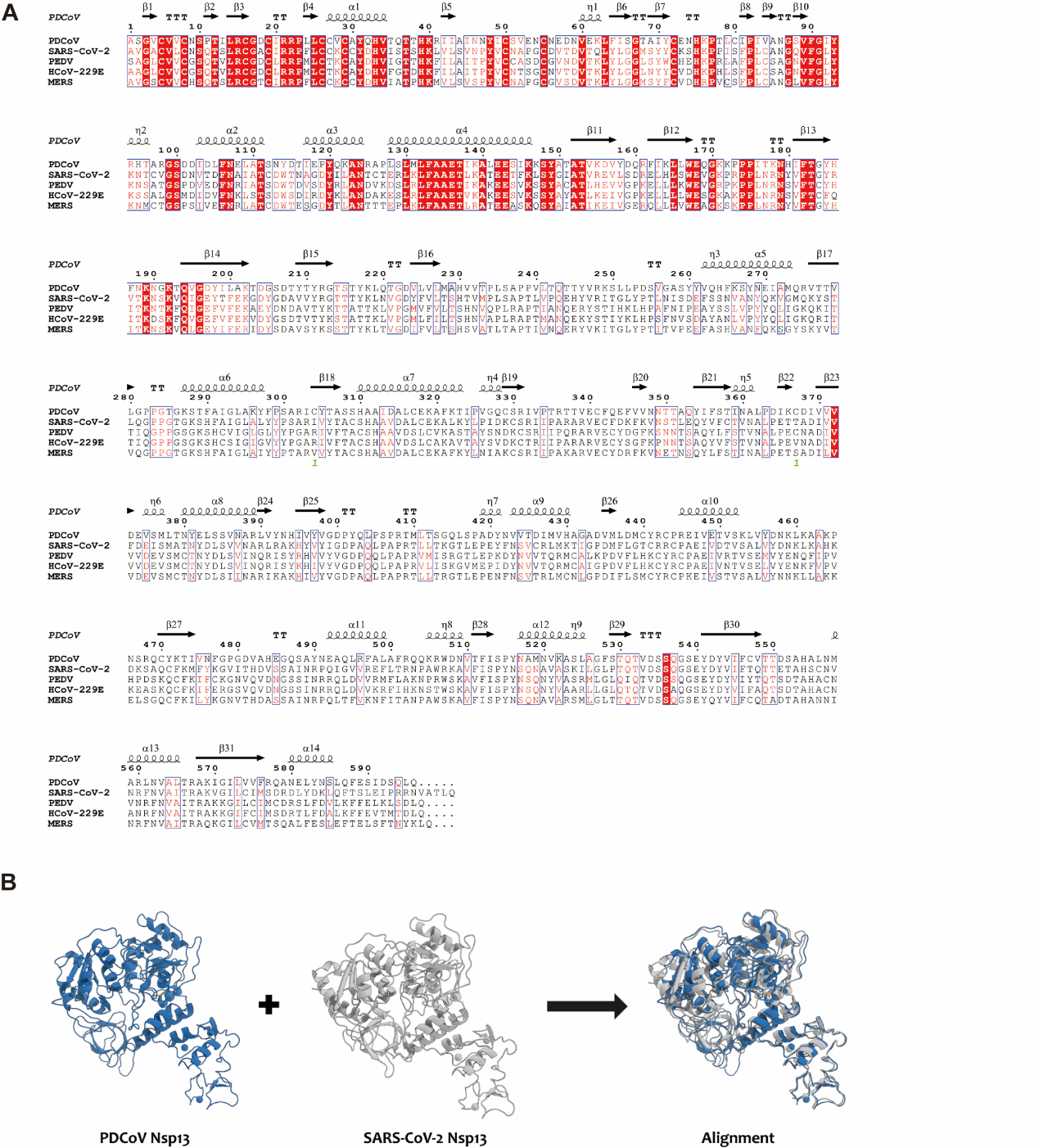


**Fig.S2: Comparative Analysis of Nsp13 Across Coronaviruses**

(A) Multiple sequence alignment of PDCoV Nsp13 with Nsp13 from other coronaviruses, including SARS-CoV-2, PEDV, HCoV-229E, and MERS-CoV.

(B) Three-dimensional structural models of PDCoV Nsp13 and SARS-CoV Nsp13 predicted using Swiss-Model.
